# Glycosaminoglycan Sulphation Affects the Seeded Misfolding of a Mutant Prion Protein

**DOI:** 10.1371/journal.pone.0012351

**Published:** 2010-08-23

**Authors:** Victoria A. Lawson, Brooke Lumicisi, Jeremy Welton, Dorothy Machalek, Katrina Gouramanis, Helen M. Klemm, James D. Stewart, Colin L. Masters, David E. Hoke, Steven J. Collins, Andrew F. Hill

**Affiliations:** 1 Department of Pathology, The University of Melbourne, Parkville, Victoria, Australia; 2 Department of Biochemistry & Molecular Biology and Bio21 Molecular Science and Biotechnology Institute, The University of Melbourne, Parkville, Victoria, Australia; 3 The Mental Health Research Institute, The University of Melbourne, Parkville, Victoria, Australia; 4 Department of Biochemistry and Molecular Biology, Monash University, Clayton, Victoria, Australia; Ohio State University, United States of America

## Abstract

**Background:**

The accumulation of protease resistant conformers of the prion protein (PrP^res^) is a key pathological feature of prion diseases. Polyanions, including RNA and glycosaminoglycans have been identified as factors that contribute to the propagation, transmission and pathogenesis of prion disease. Recent studies have suggested that the contribution of these cofactors to prion propagation may be species specific.

**Methodology/Principal Finding:**

In this study a cell-free assay was used to investigate the molecular basis of polyanion stimulated PrP^res^ formation using brain tissue or cell line derived murine PrP. Enzymatic depletion of endogenous nucleic acids or heparan sulphate (HS) from the PrP^C^ substrate was found to specifically prevent PrP^res^ formation seeded by mouse derived PrP^Sc^. Modification of the negative charge afforded by the sulphation of glycosaminoglycans increased the ability of a familial PrP mutant to act as a substrate for PrP^res^ formation, while having no effect on PrP^res^ formed by wildtype PrP. This difference may be due to the observed differences in the binding of wild type and mutant PrP for glycosaminoglycans.

**Conclusions/Significance:**

Cofactor requirements for PrP^res^ formation are host species and prion strain specific and affected by disease associated mutations of the prion protein. This may explain both species and strain dependent propagation characteristics and provide insights into the underlying mechanisms of familial prion disease. It further highlights the challenge of designing effective therapeutics against a disease which effects a range of mammalian species, caused by range of aetiologies and prion strains.

## Introduction

Transmissible spongiform encephalopathies (TSE) or prion diseases are a group of invariably fatal neurodegenerative disorders associated with misfolded conformers (PrP^Sc^) of the normal cellular prion protein (PrP^C^). In animals the disease occurs naturally as scrapie in sheep, bovine spongiform encephalopathy (BSE) in cattle and chronic wasting disease (CWD) in deer and elk. In humans the disease occurs in sporadic, familial and acquired forms with phenotypes including Creutzfeldt-Jakob Disease, Gerstmann-Sträussler-Scheinker syndrome (GSS) and Fatal Familial Insomnia [1]. The transmissible nature of prion disease has been attributed to the template directed misfolding of PrP^C^, which is supported by the absolute requirement of PrP^C^ expression for disease transmission and pathogenesis [2]. The protein only hypothesis proposes that PrP^Sc^ is the principal component of this infectious agent or template [3]. However, it is not clear whether PrP^Sc^ is the only component of the infectious and/or pathogenic entity.

Cell-free models of template directed PrP^C^ misfolding (or conversion to PrP^Sc^) have demonstrated that PrP^Sc^ can induce a conformational change in PrP^C^, rendering it protease resistant (referred to as PrP^res^) [4], [5], [6] and infectious under prescribed conditions [7]. Previously, the efficiency of this process using partially purified constituents has been low, often requiring a large excess of PrP^Sc^, which has been proposed to reflect the need for a catalytic co-factor in the process [8], [9]. This view is further supported by the low levels of infectivity produced by folding recombinant PrP into a protease resistant form, although this may also reflect the absence of post-translational modification of the recombinant protein and the nature of the transgenic mouse model used in the bioassay [9], [10].

The reported ability of polyanions to stimulate the misfolding of partially purified mammalian or recombinant PrP^C^ and generate infectivity in the absence of an initiating PrP^Sc^ seed provides compelling evidence for the role of a cofactor for the acquisition of prion infectivity [11], [12]. Negatively charged macromolecules or polyanions, including nucleic acids [11], [12], [13], [14], [15], [16], [17], [18], [19], [20], phospholipids [21], [22], [23], [24] and glycosaminoglycans (GAGs) have been implicated as facilitating cofactors in the conversion of PrP^C^ to PrP^Sc^ and thereby in the transmission and pathogenesis of prion disease. Mechanistically, GAGs have been proposed to act as scaffolds to support the misfolding of PrP^C^ [25]. Further, GAGs have been reported to act as receptors for PrP^Sc^ on the cell surface [26], [27], affect PrP^C^ trafficking [28], [29], [30] and are also found in PrP^Sc^ associated plaques [31], [32]. Treatments, which modify the GAG content of prion infected cells, or treatment of infected cells with GAGs (or GAG mimetics) have been shown to clear prion infection [28], [33]. Pentosan polysulphate (PPS), a heparan sulphate mimetic, can prolong incubation time in prion infected mice [34] and is currently being used on a compassionate basis in variant CJD [35], [36]. Significantly, unlike RNA, GAGs are found at the cell surface and along the endosomal pathway where PrP^Sc^ formation has been proposed to occur [25].

Whilst the ability of polyanions to stimulate PrP^res^ formation in cell-free assays and from recombinant PrP appears to be species independent [14], [37], [38], [39], PrP^res^ formation following the specific depletion of polyanions from the PrP^C^ substrate appears to be host species specific [40]. Using a cell-free model to investigate reaction conditions and cofactors affecting the susceptibility of a murine PrP^C^ substrate to seeded PrP^res^ formation, we report here that PrP^res^ formation is significantly and specifically inhibited by the degradation of endogenous nucleic acids or heparan sulphate. We further show that treatment to modify the degree of GAG sulphation has a differential effect on the ability of wild-type PrP and PrP encoding a mutation associated with familial prion disease to act as a substrate for conversion to PrP^res^. This may be attributed to the differing ability of wild-type and mutant PrP^C^ to bind to GAGs, suggesting that cellular cofactors differentially modulate sporadic and familial forms of prion disease and implicates subtle changes in the GAG repertoire in the pathogenesis of prion disease.

## Results

### Heparan sulphate and electrostatic involvement in cell free PrP^res^ formation

**Figure 1:**
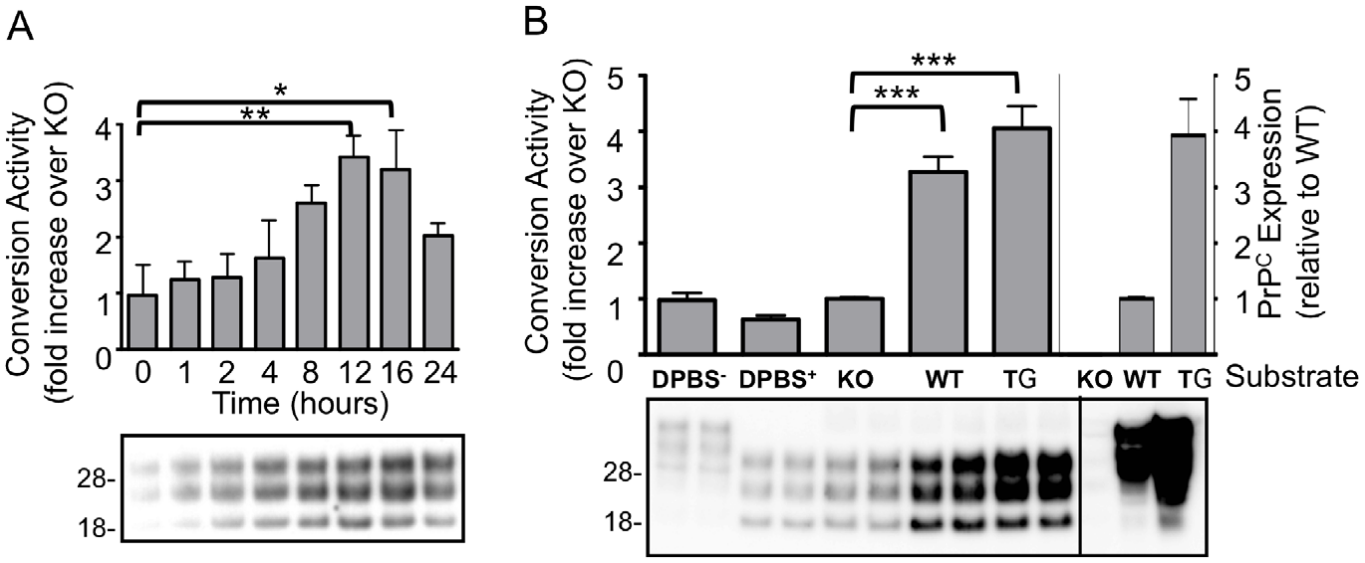
Conversion activity of brain derived PrP^C^ in the CAA seeded with infected brain homogenates. (A) UBH from balb/c (WT) mice were subjected to the CAA in the presence of IBH for differing periods of time (0–24 hours). B) The CAA was performed for 16 hours using IBH added to DPBS, or UBH from KO, WT or PrP over expressing Tga20 (TG) mice. DPBS represents total (DPBS^−^ without PK treatment), and protease resistant (DPBS^+^ with PK treatment) PrP present in the IBH used to seed the CAA. Relative PrP^C^ expression (without PK treatment) is shown in right of panel for KO, WT and TG mice. Conversion activity was determined as the fold increase in immunoreactive signal of WT relative to KO reactions after overnight (or as indicated) incubation at 37°C and treatment with PK (100µg/ml, 1hr at 37°C). Blots developed with 03R19. Molecular weights (kDa) are shown. Western blots are representative of replicated experiments, quantification is based on at least three experiments, mean and SEM are shown. *p<0.05, **p<0.01, ***p<0.001 using one-way analysis of variance (ANOVA) with Tukey's multiple comparison test (GraphPad, Prism).

The Conversion Activity Assay (CAA) generates PrP^res^ from a PrP^C^ substrate derived from an uninfected brain homogenate (UBH) seeded with a prion infected brain homogenate (IBH). Using the M1000 mouse adapted prion strain [41] as the IBH seed, PrP^res^ formation occurs in a time (Figure 1A) and PrP^C^ dependent manner with PrP^res^ generated from the balb/c (WT) but not Prnp^−/−^ (KO) mouse brain homogenates (Figure 1B). While the PrP^C^ contained within the WT UBH was efficiently converted, there is evidence of further limiting, non-PrP factors in the process as only a small proportion of the available PrP^C^ substrate (24±9%, n = 8) is converted in the reaction using UBH derived from PrP^C^ over expressing Tga20 mice [42]. That an increase in PrP^C^ does not significantly increase conversion efficiency suggests that factors other than PrP^C^ in the UBH may limit the output of the assay (Figure 1B).

**Figure 2:**
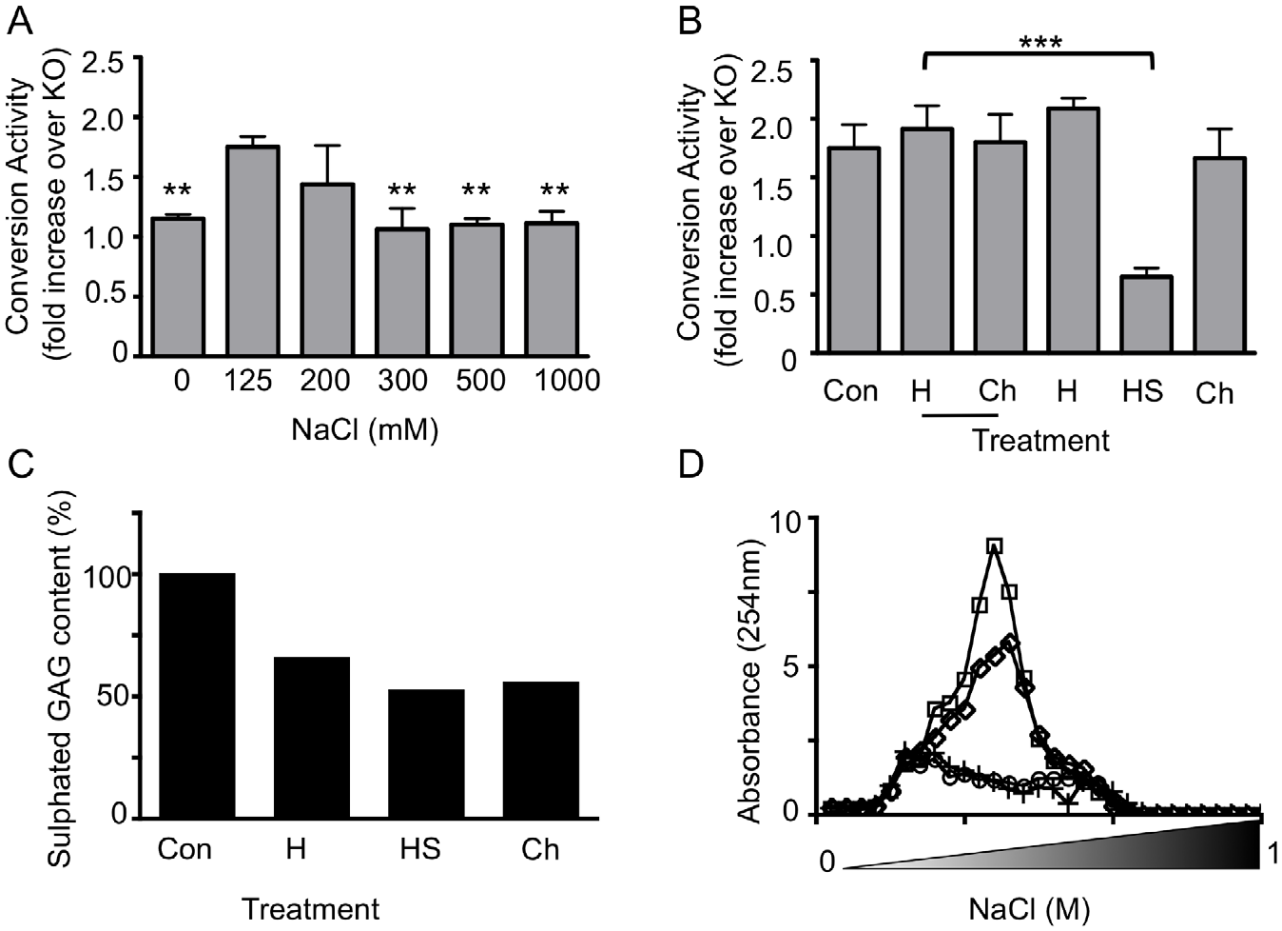
Conversion activity of brain derived PrP^C^ in the CAA seeded with infected brain homogenates is sensitive to ionic strength and inhibited by the specific depletion of heparan sulphate. (A) The CAA was performed using IBH diluted in UBH prepared from WT and KO mice in Tris-HCl pH 7.4 and the indicated concentrations of NaCl. ** Indicates a significant reduction in conversion activity relative to 125mM NaCl. B) The CAA was performed using IBH diluted in UBH prepared from WT mice in 125mM NaCl/Tris-HCl pH 7.4 after treatment with Heparinase I (H), Heparinase III (HS), Chondroitinase ABC (Ch), their corresponding buffers (underlined) or without treatment (Con). Conversion activity was determined as the fold increase in immunoreactive signal of WT relative to KO reactions after overnight incubation at 37°C and treatment with PK (100µg/ml, 1hr at 37°C). Quantification (A, B) is based on at least three experiments, mean and SEM are shown. **p<0.01, ***p<0.001 using one-way analysis of variance (ANOVA) with Tukey's multiple comparison test (GraphPad, Prism). C) The amount of sGAG purified from UBH treated with Heparinase I (H), Heparinase III (HS) and Chondrotinase ABC (Ch) or untreated (Con) was determined by Blyscan analysis and normalised to the amount of sGAG recovered from buffer controls (not shown). D) The absorbance (254nm) of sGAG eluted from a Q-Sepharose HiTrap anion exchange column in increasing concentrations of NaCl (0–1M). GAGs were purified from control (□), Heparinase I treated (⋄) and Heparinase III treated (○) or Chondroitinase ABC treated (+) brain homogenates. Quantification (C, D) is based on an analysis performed in duplicate.

Electrostatic forces mediate many biological interactions [43], [44], [45] and have been reported to affect the folding and stability of PrP [46]. To investigate whether electrostatic forces play a role in the cell-free formation of PrP^res^ the CAA was performed in buffers of increasing ionic strength (Figure 2A). Using a similar assay, the ability of IBH derived PrP^Sc^ to drive the amplification of PrP^res^ has been shown to decrease in the absence of NaCl [47]. However, the interaction was also significantly reduced in high ionic strength buffers (≥300mM; p<0.01 one-way ANOVA analysis relative to125mM NaCl), consistent with a physiologically relevant interaction and implicating electrostatic interactions in the seeded formation of PrP^res^.

Electrostatic interactions may exist between polyanionic molecules, such as sulphated GAG (sGAG) species and the polybasic regions of PrP [48], [49], [50]. The contribution of sGAG to PrP^res^ formation using the CAA described here was investigated by specific depletion of the endogenous sGAG content of the UBH used as the PrP^C^ substrate in the CAA (Figure 2B). Following optimisation of the conditions required for efficient sGAG digestion, the presence of sulphated species in the UBH was decreased (Figure 2C) and a reduction of polysaccharide chains shown by decreased absorbance of purified GAGs separated using an anion exchange column (Figure 2D). The capacity of the UBH to act as a conversion substrate in the CAA was specifically and significantly (p<0.001) reduced following heparinase III treatment to preferentially degrade heparan sulphate but not other sulphated GAG species, including heparin and chondroitin sulphate species. Treatment to deplete GAGs from the substrate did not reduce the amount of available PrP^C^ substrate (data not shown).

**Figure 3:**
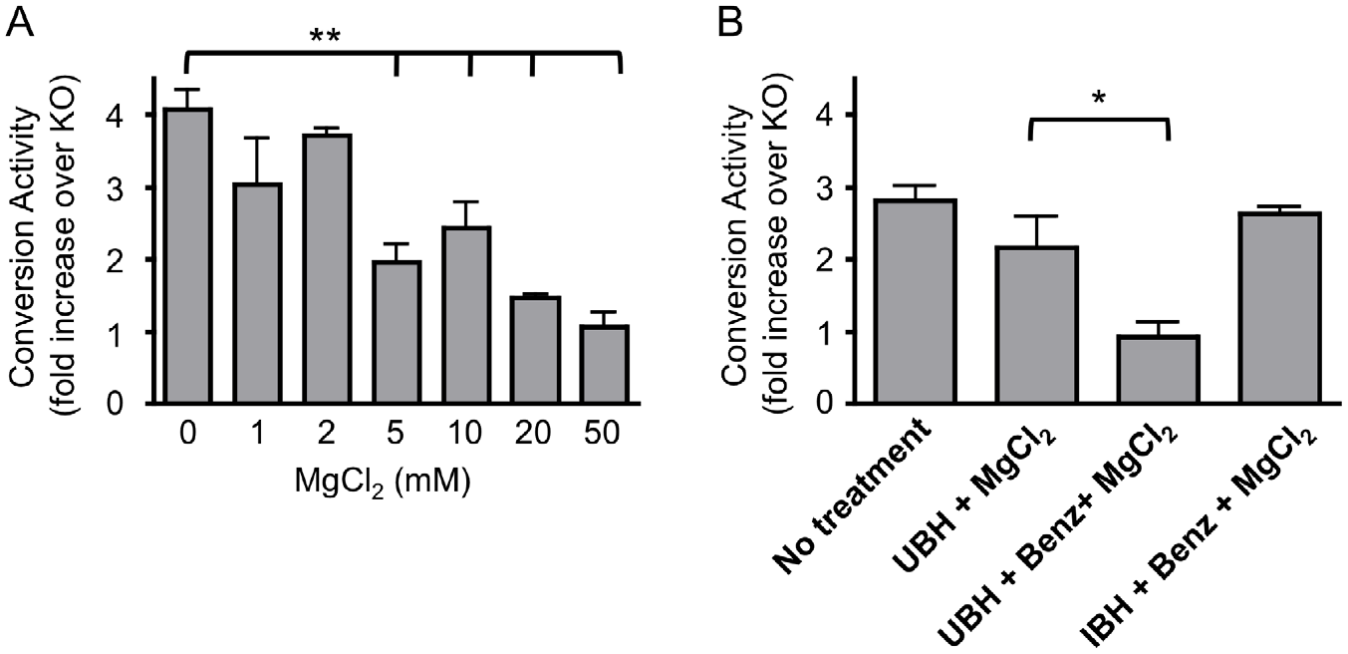
Conversion activity in the CAA following Benzonase treatment of UBH or IBH. A) The CAA was performed in the presence of the indicated concentrations of MgCl_2_ and B) performed following pre-treatment as indicated. Conversion activity was determined as the fold increase in immunoreactive signal of treated samples relative to their equivalent KO reactions after overnight incubation at 37°C and treatment with PK (100µg/ml, 1hr at 37°C). Quantification is based on at least three experiments, mean and SEM are shown. **p<0.001 or *p<0.05 using one-way analysis of variance (ANOVA) with Dunnet's test for multiple comparisons against the indicated control (GraphPad, Prism).

It has been previously reported that the conversion activity of 263K, a hamster adapted sheep scrapie strain, is decreased by enzymatic treatment to reduce the nucleic acid content [14] and recently suggested that nucleic acids do not contribute to the conversion activity of mouse adapted prion strains [40]. To determine if this is true of all mouse prion strains the CAA was performed using the M1000 strain of mouse adapted human prions. The effect of MgCl_2_ concentration, a divalent cation required for the efficient activity of the nucleic acid digesting enzyme, Benzonase was first investigated to ensure that the effect of the treatment was enzyme specific (Figure 3A). It was found that concentrations of MgCl_2_ required for optimal activity of Benzonase (1–2mM) did not significantly affect conversion activity, while concentrations at or over 5mM significantly decreased conversion activity. Benzonase treatment of the mouse derived UBH, significantly decreased conversion activity, relative to the buffer control, whereas pre-treatment of the IBH seed of the CAA had no effect (Figure 3B). This suggests that nucleic acids present in the UBH substrate, but not the IBH seed, can act as catalysts or scaffolds for PrP^res^ formation.

### Familial prion disease mutations affect sGAG binding and conversion activity of PrP^C^ in the CAA

**Figure 4:**
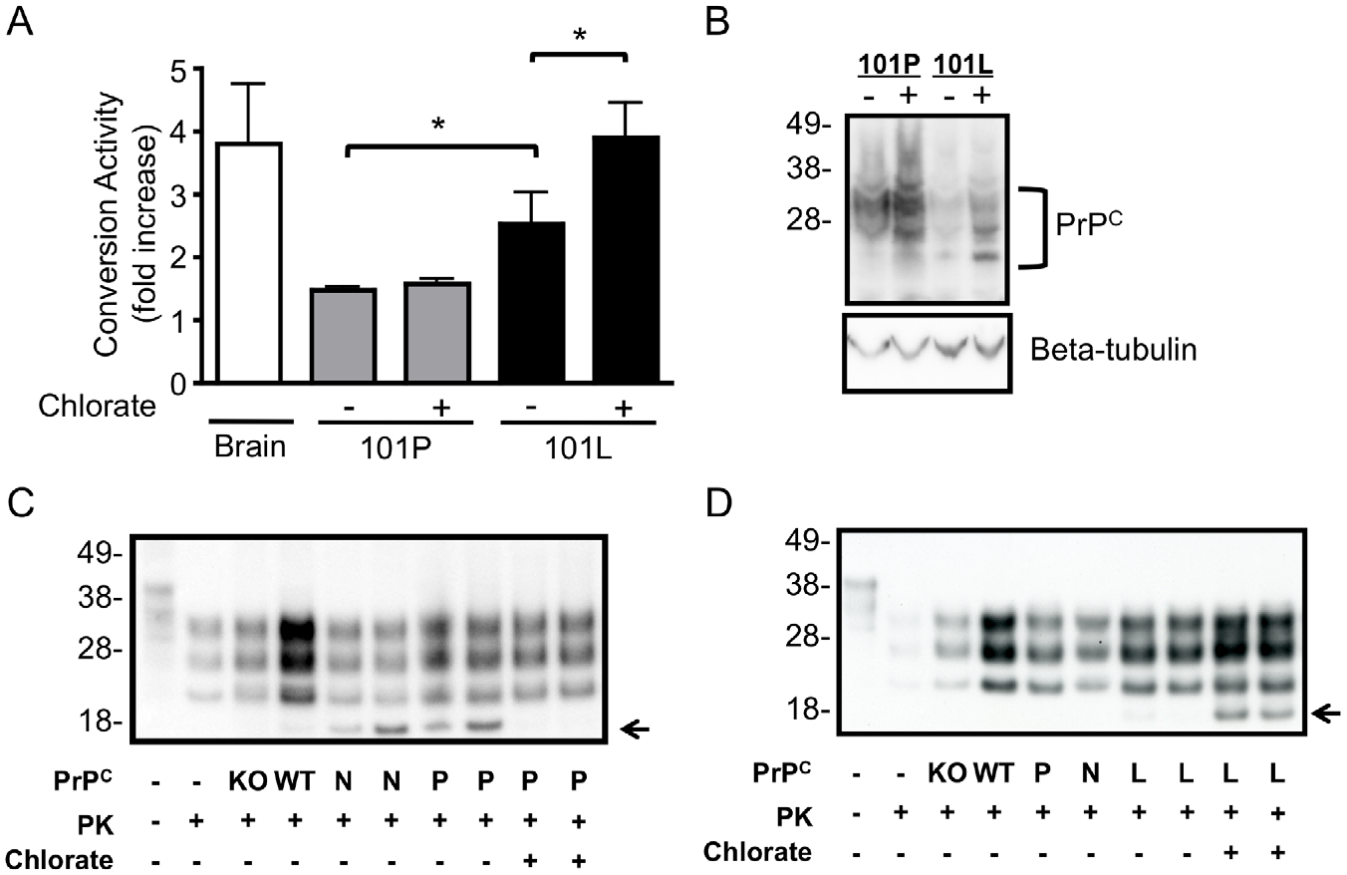
Conversion activity of wild-type and mutant PrP^C^ expressed in RK-13 cells in the CAA following chlorate treatment to modify the sulphation of GAG. The CAA was performed using lysates prepared from RK-13 cells expressing WT (101P) and mutant (101L) moPrP. A) Quantification of conversion activity of 101P and 101L moPrP left untreated (−) or treated with 30mM chlorate (+) or UBH (Brain). B) Western blot analysis of PrP^C^ expression in 101P and 101L moPrP left untreated (−) or treated with 30mM chlorate (+). Equivalent protein loaded in each lane, blots probed with beta-tubulin. CAA performed using (C) 101P-moPrP and (D) 101L-moPrP cells left untreated (−) or treated with 30mM chlorate (+). Conversion activity was determined as the fold increase in immunoreactive signal relative to puroRK reactions after overnight incubation at 37°C and treatment with PK (100µg/ml, 1hour at 37°C). Blots developed with 03R19. Molecular weight (kDa) is shown. Western blots are representative of replicated experiments, quantification is based on at least three experiments, mean and SEM are shown. *p<0.05 two-tailed t-test of indicated pairs. In (C) and (D) CAA performed using KO and WT mouse brain homogenates (with quantitation shown as brain in A) and cell lysate derived from puroRK (N), 101P (P) and 101L (L) moPrP expressing cell lines. Truncated fragment (←) was not a consistently observed in either wildtype or mutant cell lines and was not included in analysis.

Mutations associated with familial prion disease located in the C-terminal region of PrP (121–231) do not reduce the stability of PrP [51]. However, the proline to leucine mutation at residue 101 of full length mouse PrP (P102L human PrP sequence) has been reported to alter the alpha-helical content of full length PrP [52] and this and other familial mutations have been reported to increase the GAG binding capacity of PrP [53]. Expression of endogenous levels of 101L mutation are not sufficient to induce spontaneous disease in knock-in mice, although the mutation does alter the susceptibility of mice to prion infection [54]. To investigate how GAGs may affect the susceptibility of the 101L mutation to undergo seeded misfolding we developed a CAA model using mouse PrP^C^ exogenously expressed in RK13 cells as the substrate (Figure 4). RK13 cells do not express detectable levels of PrP, but become susceptible to infection by mouse adapted prion strains through exogenous expression of mouse PrP [55], [56], [57]. When lysates of mouse PrP^C^ expressing cells were used as the substrate in the CAA a significant increase in PrP^res^ was detected relative to RK-13 cells that had been transfected with the empty expression vector (puroRK). This exogenous expression system also enabled the investigation of the conversion activity of moPrP harbouring a P101L mutation (Figure 4A). The conversion activity of reactions containing mutant 101L-moPrP was significantly greater than those of wild-type 101P-moPrP, despite detection of lower 101L-moPrP levels (Figure 4B).

To investigate whether the association of sGAG with PrP^C^ affects the conversion process wild-type 101P-moPrP and mutant 101L-moPrP cells were treated with chlorate, a general inhibitor of GAG sulphation [58], and PrP^C^ formed in the presence of modified GAG sulphation used as substrate in the CAA. The conversion activity of wildtype 101P-moPrP was not significantly affected by chlorate treatment of the cells (Figure 4A,C). In contrast the conversion activity of mutant 101L-moPrP was significantly increased following chlorate treatment (Figure 4A,D).

**Figure 5:**
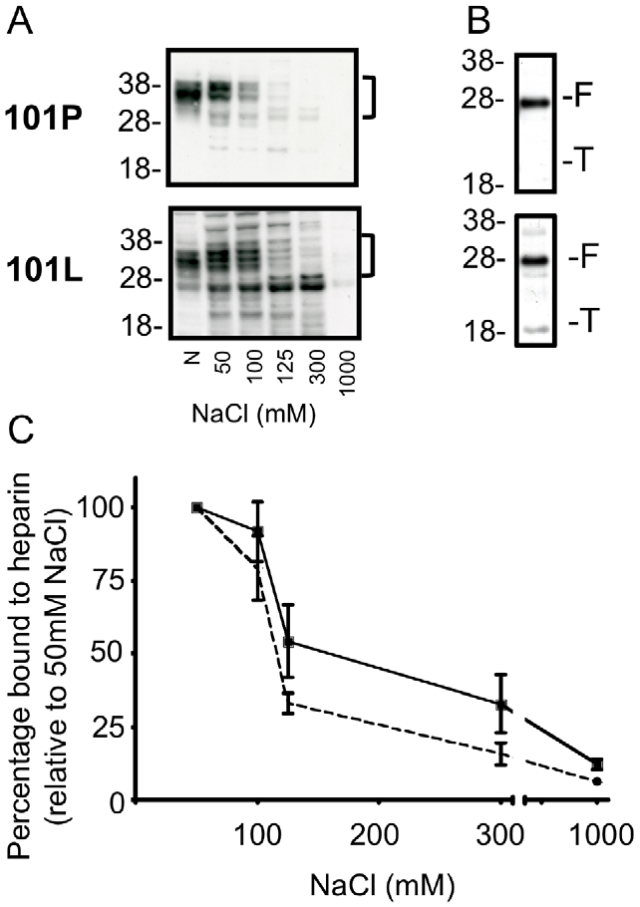
Binding of wild-type and mutant PrP^C^ expressed in RK-13 to sGAG. A) The ability of wildtype (101P) and mutant (101L) moPrP expressed in RK-13 cells to bind heparin sepharose beads in the presence of increasing concentrations of NaCl (50, 100, 125, 300, 1000mM), was determined by western blot analysis. N shows neat input. B) PrP^C^ bound to heparin sepharose in the presence of 50mM NaCl was treated with PNGaseF before western blot analysis. Full length (F) and truncated (T) PrP species are shown. C) The percentage of 101P (dotted line) and 101L (solid line) moPrP bound to heparin sepharose in increasing concentrations of NaCl was determined relative to binding in 50mM NaCl. Blots developed with 03R19. Molecular weight (kDa) is shown. Western blots are representative of replicated experiments, quantification is based on at least three experiments, mean and SEM are shown. Binding differed significantly by Two-way ANOVA (p<0.001).

To understand the different response of 101P and 101L moPrP to chlorate treatment we investigated their relative GAG binding capacities. The heparin binding capacity of 101L-moPrP was significantly greater (p<0.001, two way ANOVA) than that of 101P-moPrP (Figure 5A, C). An N-terminally truncated form of PrP does not appreciably bind to sGAG [48], consistent with a GAG binding site in the N-terminal region of PrP [49], [50]. Mutations associated with familial prion disease have been shown to reveal a cryptic GAG binding site down stream of the residue 90, which may enable the C2 fragment of PrP to bind to GAGs [53]. PNGaseF treatment revealed a truncated fragment of 101L-moPrP bound to heparin whereas the same fragment present in the 101P-moPrP expressing cells was not detected (Figure 5B). This fragment was detected using antibodies 03R19 but not with the N-terminal antibody 03R17 indicating that it lacked N-terminal residues 23–30 (data not shown).

## Discussion

Large negatively charged macromolecules (GAGs, nucleic acids and phospholipids) have been implicated in the pathogenesis of prion diseases. Nucleic acids, and in particular RNA has been identified as a potential co-factor in the formation of the disease associated isoform of the prion protein, in hamster [11], [14] but not mouse models of prion disease [40]. The current study using a mouse adapted human prion strain, provides further insight into the prion strain and species specific requirements for prion propagation. We report that depletion of either nucleic acids or sGAG and in particular heparan sulphate, prevent the cell free formation of PrP^res^ from murine PrP^C^ seeded with mouse derived PrP^Sc^. Changes to GAG sulphation through chlorate treatment increased the ability of PrP^C^ encoding the P101L mutation linked with familial prion disease to form PrP^res^, which may be related to the ability of this molecule to associate with under sulphated GAG species.

Recent reports have suggested that the cofactors required for efficient hamster PrP^res^ formation may be species specific as the depletion of RNA from a murine derived substrate did not affect PrP^res^ formation [14], [40]. In contrast prion infectivity can been generated from either hamster PrP or recombinant murine PrP, in the absence of a PrP^Sc^ seed, by the addition of RNA, albeit in the presence of lipids [11], [12]. Thus it would appear that RNA stimulation of de novo PrP^res^ formation is species independent. Furthermore the data presented here showing that depletion of nucleic acids from the PrP^C^ substrate of a mouse adapted model of human prion disease prevented PrP^res^ formation, indicates that the requirement for endogenous RNA may be prion strain dependent. Moreover the use of a prion strain originally derived from a patient with GSS in the current study rather than scrapie or bovine spongiform encephalopathy strains raises the possibility that human prion strains have different cofactor requirements to animal prion strains. We are investigating this possibility further using a mouse adapted prion strain developed from a T2MM sporadic prion strain [56].

A further species or strain specific effect is raised by the significant and specific effect of heparan sulphate depletion on conversion activity shown here. Unlike earlier reports, in which the heparinase III treatment did not affect the conversion activity of a hamster PrP^C^ substrate seeded with 263K hamster adapted scrapie brain homogenate [14], we show that specific depletion of endogenous heparan sulphate inhibits the conversion activity of a mouse PrP^C^ substrate when seeded with the M1000 mouse adapted human prion strain. The curing effect of heparinase III, but not heparinase I and chondroitinase ABC treatment of prion infected N2a cells has been previously reported and proposed to relate to either the relative GAG content of N2a cells or relate to the cleavage specificity of the enzymes [30]. In particular it was suggested that prion propagation might require under sulphated GAGs that are the target of heparinase III or relate to the length of the stub that survives enzymatic treatment. Our results are consistent with a role for specific GAG sulphation patterns in the conversion process.

In the model studied here either depletion of nucleic acids or heparan sulphate led to the complete abolition of conversion activity. The apparent requirement for two cofactors in PrP^res^ formation is not unexpected as the formation of infectivity from recombinant murine PrP required both RNA and lipids and the presence of lipids in infectivity derived from RNA stimulated mammalian PrP could not be excluded [11], [12]. Glycosaminoglycans and nucleic acids are both large negatively charged molecules based on an underlying carbohydrate backbone. We considered whether the high concentrations of Benzonase used to digest the nucleic acids as described here and in other reports (1000 times the manufacturers' recommended levels for general digestion of nucleic acids) may have non-specifically affected the GAG content of the substrate. However, in preliminary experiments to investigate this possibility we found no definitive change in the sulphated GAG content of homogenates following Benzonase treatment. Therefore for this strain at least both RNA and heparan sulphate are absolutely required for PrP^res^ formation.

Point mutations (including the P102L human PrP mutation, equivalent to the P101L mouse PrP mutation studied here) and octapeptide repeat expansions associated with familial prion disease increase the association of recombinant PrP with sGAGs [53]. Using PrP^C^ expressed in a mammalian cell line capable of supporting prion infection [55], [56], [57] it was shown that the affinity of 101L-moPrP for heparin was significantly increased relative to wild type moPrP. This confirms that the increased affinity of recombinant PrP encoding familial mutations for heparin [53] is also observed for PrP expressed in mammalian systems.

The 101L-moPrP mutation was more susceptible to conversion to PrP^res^ in the CAA than the wild type 101P-moPrP. As previously reported [59], introduction of the 101L mutation increased the protease resistance and insolubility of PrP expressed in RK13 cells (Welton and Lawson unpublished observations). Introduction of the same mutation does not alter the stability [60] or confer protease resistance [52] on recombinant PrP produced in a cofactor free environment, although the alpha helical content of the protein is decreased. The alpha helical content of purified PrP is decreased by binding to PPS, which has been proposed to increase the susceptibility of the protein to conversion in cell free assays by reducing the transition barrier [38]. We therefore propose that subtle conformational changes associated with the 101L-moPrP [52] result in an increase in the proportion and affinity of the 101L-moPrP population for a binding partner (present in a mammalian expression system) and increase its ability to convert to the PrP^res^ form. An alternative possibility not investigated here is the origin of the M1000 strain from a patient with GSS associated with the P102L mutation [61], [62]. Although adapted to mice and therefore on a wild type moPrP background we cannot exclude the possibility that the PrP^Sc^ from the original prion strain preferentially converts PrP^C^ encoding the original mutation. It may also reflect a faster replication kinetics as has been reported for PrP encoding octapeptide repeat insertions in a cell-free conversion assay [63].

Both the P102L and E220K mutations associated with familial prion disease do not require residues 23–27 for GAG binding, with binding of mutant PrP^C^ mediated through a cryptic GAG binding site located between residues 109–136 [53]. Consistent with this we report the increased binding affinity of 101L-moPrP for heparin and the preferential binding of a 22kDa fragment consistent with C2 (89–230) from 101L but not 101P moPrP. The association of PrP with GAGs through this alternative-binding domain may play a role in the pathogenic process.

It was surprising that modification of GAG sulphation with chlorate did not decrease the conversion activity of moPrP. Chlorate treatment does not change the PK-resistance or solubility of wild type 101P-moPrP (Welton and Lawson unpublished), although it did increase the PrP levels, perhaps by altering the metabolism of PrP^C^ [29]. Chlorate competitively inhibits the formation of the sulphate donor 3′-phosphoadenosine 5′-phosphate (PAPS) required for GAG sulphation. When cells are grown in medium containing normal sulphate supplementation, as performed here, sulphation of heparan sulphate is selectively inhibited, with 6-O-sulphation inhibited before 2-O-sulphation [64]. Previous studies have highlighted the importance of 2-O but not 6-O sulphation for the interaction of wildtype PrP with heparin [49] and the role of under sulphated GAGs in prion propagation [30]. Therefore it is possible that under the conditions used in this study sulphation required for the interaction of wild type 101P-moPrP with sGAG remained unaltered. Whereas due to the altered GAG binding pattern of 101L-moPrP, selective inhibition of sulphation may have increased the profile of GAGs that could bind and facilitate the conversion of mutant 101L-moPrP. Intriguingly, chlorate increased the solubility of mutant 101L-moPrP (Welton and Lawson unpublished observations), which may have affected the ability of this species to be converted to PrP^res^.

This study has revealed a further complexity to the role of cofactors in the propagation of prions. Although prion infectivity can be generated from PrP in the absence of cofactors it appears that the addition of cofactors may augment the conversion process [9], [10]. This may explain both species and strain dependent propagation characteristics and provide insights into the underlying mechanisms familial prion disease. It further highlights the challenge of designing effective therapeutics against a disease which effects a range of mammalian species, caused by range of aetiologies and prion strains.

## Materials and Methods

### Ethics statement

The use of tissue sourced from prion infected (AEEC#04154, 0707227) and uninfected (AEEC#05090, 0810787) mice was approved for this study by the University of Melbourne Animal Ethics Committee

### Preparation of prion infected brain homogenates (IBH)

Brains were collected from balb/c mice in the terminal stage of disease following intracerebral inoculation with M1000 prions [41]. For use as a seed in the cell free assay of PrP^res^ formation (Conversion Activity Assay described below), 10% (w/v) brain homogenates were prepared in calcium and magnesium free Dulbecco's phosphate buffered saline (DPBS) or 20 mM Tris-HCl pH 7.4 supplemented with 1% (v/v) Triton-X 100. Homogenates were prepared by passing tissue through a graded series of needles (18G, 21G, 24G, 26G). The final sample was then cleared at 200×g for 2 minutes, the supernatant snap frozen in liquid nitrogen and stored at −80°C.

### Preparation of uninfected brain homogenates (UBH)

Brain tissues were collected from wild type (balb/c), Prnp knock out (Prnp^−/−^; [2]) and Prnp overexpressing (Tga20; [42]) mice. Brain homogenates (10% w/v) were prepared in DPBS or 20mM Tris-HCl pH 7.4 supplemented with EDTA-free *e*complete mini protease inhibitors (PI; Roche) using a graded series of needles as described above.

### GAG lyase treatment of UBH

GAG specific lyases were obtained from Sigma. Heparinase I and Heparinase III from *Flavobacterium heparnium* were prepared in 10 mM Tris-HCl pH 7.4, 4 mM CaCl_2_, 50mM NaCl. Chondroitinase ABC from *Proteus vulgaris* was prepared in 0.01% (w/v) BSA. Reconstituted lyases were stored at −80°C.

UBH (50% w/v) prepared from 100 mg balb/c or Prnp^−/−^ mice in 10 mM Tris-HCl pH 7.4 were treated with GAG lyase (Heparinase I and III 20U/100 mg wet tissue equivalents; Chondroitinase ABC 8U/100 mg wet tissue equivalents) or dilution buffer (Heparinase Buffer, 10 mM Tris-HCl pH 7.4, 4 mM CaCl_2_, 50 mM NaCl; Chondroitinase Buffer 0.01% (w/v) BSA) for 4 hours at 37°C with agitation.

Following treatment, 40 mg wet tissue equivalents was diluted to 10% (w/v) in 20 mM Tris-HCl pH 7.4 and 125 mM NaCl (final concentration), snap frozen in liquid nitrogen and stored at −80°C for subsequent use in the cell-free conversion activity assay (CAA). GAGs were purified from the remaining 60 mg wet tissue equivalents as described previously [65]. Briefly homogenates were delipidated at room temperature for 2 hours in 4 volumes 1∶2 chloroform: methanol (v/v). After centrifugation (3,000×g, 10 minutes) the pellet was dissolved in ethanol at a ratio of 1.5mL/g initial tissue equivalents to remove organic solvents. After centrifugation (3,000×g, 10 minutes) the pellets were dried overnight at 37°C and suspended at 1.5mL/g initial tissue equivalents in 0.1 M Tris-HCl pH 8.0, 1mM CaCl_2_ and treated with 10 mg/mL pronase (pre-incubated for 30 minutes at 60°C to eliminate exogenous glycosidase activity, Sigma) for 72 hours at 37°C by adding 40µl of enzyme at 0, 24 and 48 hours. The preparation was then treated with Benzonase (0.25U/mL, Progen) for 18 hours at 37°C, followed by 40µl pronase for a further 24 hours at 60C°. O-linked carbohydrates were released from the remaining peptides by titration with 10mM NaOH to pH 10–11 before the addition NaBH_4_ (1M) and incubation for 16 hours at 45°C. Samples were neutralized by the addition of acetic acid and then centrifuged at 4,000×g for 15 minutes. The supernatant was recovered and the pellet washed a further two times in purified water, with the wash supernatant added to initial supernatant. After the addition of 2 volumes of acetone the resulting precipitate (4°C, for 24 hours) was recovered by centrifugation at 4,000×g for 15 minutes and was air dried and dissolved in 1mL purified water.

Purified GAGs were analyzed for their sulphated GAG content by Blyscan analysis (Bicolor Ltd) as per the manufacturers instructions using Chondroitin sulphate C from shark cartilage (Sigma) to generate a standard curve. Samples were analyzed in triplicate using a microplate reader at a wavelength of 650nm.

Further purification of GAG species was obtained by separation on a 0.7×2.5cm Q-Sepharose HiTrap anion exchange column (GE Healthcare). The column was prepared with 5 column volumes of binding buffer (20mM Tris-HCl pH 7.4). Purified GAGs were injected onto the column (1mL/minute) and washed with 5 volumes of binding buffer to remove unbound molecules before bound GAGs were eluted using a continuous salt gradient (0–1M NaCl in binding buffer), 1.5mL fractions were collected at a flow rate of 1mL/min and elution measured at an absorbance wavelength of 256nm.

### Benzonase treatment of brain homogenates

UBH and IBH prepared in DPBS were treated with Benzonase (Merck, 1.2 mU/ml, 10 mM MgCl_2_, 5 minutes at 37°C). IBH were then diluted 1 in 50 in DPBS/1% (v/v) TX-100 before use in the Conversion Activity Assay. UBH were diluted ½ by the addition of IBH in the Conversion Activity Assay.

### Generation of RK13 cells expressing wildtype and mutant mouse PrP

Wildtype mouse PrP coding sequence was cloned into the pIRESpuro2 vector (Clontech) and verified by DNA sequencing [57]. The P101L mutation encoding the mouse sequence equivalent of the P102L mutation in human PrP was generated from wild-type mouse PrP cloned into the pIRESpuro2 vector using the Quikchange II site-directed mutagenesis kit (Stratagene) following a modified protocol. Briefly, primers (forward, 5′CCATAATCAgTggAACAAgCTCAgCAAACCAAAAACC 3′, reverse 5′ ggTTTTTggTTTgCTgAgCTTgTTCCACTgATTATgg 3′) were designed to introduce a mismatch at residue 305, resulting in the coding of a leucine at codon 101 instead of a proline. Thermocycling was modified from the manufactures guidelines and consisted of denaturation at 95°C for 50 seconds, followed by 18 cycles of denaturation at 95°C for 50 seconds, primer annealing at 60°C for 50 seconds and elongation at 68°C for 12 minutes. The reaction was held at 68°C for 7 minutes. The vector was then transformed and expressed as per the manufacturers directions. Mutations were confirmed by DNA sequencing.

The constructs were transfected into RK13 cells [66], [67] using Lipofectamine2000 (Invitrogen) and stably transfected populations of cells were selected for with puromycin (2.5µg/ml) and designated 101P-moPrP (wildtype mo-RK13) or 101L-moPrP (mutant mo-RK13). RK13 cells transfected with the pIRESpuro2 vector alone were used as negative controls and designated puroRK. Cell lines were maintained at 37°C in 5% CO_2_ in DMEM (Invitrogen) supplemented with 10% (v/v) heat-inactivated fetal calf serum (FCS) (Invitrogen), penicillin-streptomycin, L-glutamine and puromycin (2.5µg/ml).

Cell lysates were prepared for use in the CAA by suspending 10^6^ cells in 50 µl DPBS + PI and subjecting to two rounds of freeze thawing to lyse membranes.

For heparin binding experiments, cell monolayers were washed twice in ice cold PBS and lysed in the flask with lysis buffer (10 mM Tris pH 8.0, 100 mM NaCl, 1% (v/v) NP-40) at 4°C. Lysates were transferred to microfuge tubes and centrifuged for 3 minutes at 2,700×g. Total protein concentration was determined by bicinchoninic acid assay (BCA; Pierce).

### Conversion Activity Assay (CAA)

To 50µl PrP^C^ substrate (UBH or cell lysate as prepared above), 50 µl of IBH, diluted a further 1/50 in the appropriate buffer supplemented with 1% (v/v) Triton X-100, was added. Where the CAA was performed in buffers of increasing ionic strength, homogenates prepared in 20mM Tris-HCl were supplemented with NaCl to give the final concentrations indicated. The samples were agitated overnight at 300 rpm, 37°C. Samples were then treated with PK (100µg/ml) for 1 hour at 37°C. The reaction was stopped by the addition of Pefabloc SC (Roche) to 4 mM and an equal volume of 2× sample buffer added to each sample. Samples were heated to 100°C before electrophoresis and western blot analysis.

### Western immunoblot analysis

Samples prepared in NuPAGE sample buffer (Invitrogen) supplemented with 3% (v/v) β-mercaptoethanol were heated to 100°C and subjected to SDS-PAGE electrophoresis using NuPAGE Novex 12% Bis Tris gels and transferred to PVDF membranes as previously described [68]. PrP was detected with a polyclonal antibody raised against residues 89–103 of mouse PrP (03R19; [56]) developed with ECL-Plus chemiluminescent reagent (GE Healthcare) and imaged using ECL Hyperfilm (GE Healthcare) or LAS-3000 imaging system (Fuji). Deglycosylation of samples before western blot analysis was performed by PNGaseF treatment as previously described [56].

### Chlorate treatment of cell lines

GAG sulphation was inhibited by sodium chlorate (Sigma) treatment of RK13 cell lines as previously described [69]. Briefly, 70% confluent cells cultured in Optimem 10% FCS were treated with 30mM sodium chlorate and maintained for 2 passages in chlorate before cells were harvested for use in the CAA. Chlorate treatment of RK-13 cells reduces the Alcian blue reactive species in the conditioned medium, which is consistent with the loss of GAG sulphation (data not shown).

### Heparin binding of cell lysate derived PrP^C^

Heparin-Sepharose 6 Fastflow beads (GE Healthcare, UK) were equilibrated in lysis buffer (10 mM Tris-HCl pH 8.0, 100 mM NaCl, 1% v/v NP-40) for 15 minutes at room temperature and resuspended to their original volume in lysis buffer. Beads were added to cell lysates prepared as described above (30µl bead preparation: 400µg total protein in a final volume of 800µl lysis buffer). In salt competition studies, lysates were prepared in the indicated concentration of NaCl before beads and lysates were incubated with agitation for 1–2 hour at room temperature and then centrifuged to pellet beads. The pellet was washed 3 times in lysis buffer and final bead pellet resuspended in 1× sample buffer and heated to 100°C before SDS-PAGE and western blot analysis.
